# The Role of Immune Checkpoint Inhibitors in Cancer Therapy

**DOI:** 10.3390/clinpract13010003

**Published:** 2022-12-27

**Authors:** Ahmed M. Basudan

**Affiliations:** Department of Clinical Laboratory Sciences, College of Applied Medical Sciences, King Saud University, Riyadh 12372, Saudi Arabia; ahmbasudan@ksu.edu.sa

**Keywords:** checkpoint inhibitors, cancer, immunotherapy, CTLA-4, PD-1, PD-L1

## Abstract

Over the years, immune checkpoint inhibitors (CPIs) have become a powerful treatment strategy in the field of cancer immunotherapy. In the last decade, the number of FDA-approved CPIs has been increasing prominently, opening new horizons for the treatment of a wide range of tumor types. Pointedly, three immune checkpoint molecules have been under extensive research, which include cytotoxic T-lymphocyte-associated protein 4 (CTLA-4) and programmed cell death protein-1 (PD-1) and its ligand-1 (PD-L1). Despite remarkable success, not all patients respond positively to therapy, which highlights the complexity of the tumor microenvironment (TME) and immune system. This has led to the identification of molecular biomarkers to predict response and toxicity. In addition, there has been an emerging focus on developing new delivery and targeting approaches for better drug efficacy and potency. In this review, we highlight the mechanism of action of major CPIs, their clinical impact, variation in effectiveness, response prediction, updated clinical indications, current challenges and limitations, promising novel approaches, and future directions.

## 1. Introduction

One of the early developments that formed the basis for cancer immunotherapy research was the concept of cancer immunosurveillance, which was introduced by Lewis Thomas and Frank Macfarlane Burnet more than five decades ago [1,2,3]. Another significant discovery was the hypothesis that T-cell activation requires a two-signal process, which was introduced in 1970 by Bretcher and Cohn, then validated later by Schwartz et al. [4,5]. In that model, the first signal involves recognition of a specific antigen by the T-cell receptor (TCR), followed by a second costimulatory interaction between a T-cell costimulatory receptor and its ligand on an antigen-presenting cell (APC). Following these discoveries, multiple mechanisms of various immunological processes in cancer recognition and elimination have been elucidated. A broad range of cosignaling (costimulatory or coinhibitory) receptors has been identified.

Advances in cancer immunotherapy have rapidly emerged in the past decade. This is acknowledged by the Nobel prize awarded in 2018 to James Allison and Tasuku Honjo for the discovery of cytotoxic T-lymphocyte-associated protein 4 (CTLA-4) and programmed cell death protein-1 and its ligand-1 (PD-1/PD-L1), respectively, in the inhibition of negative immune regulation [6]. This review sheds light on a specific class of immunotherapy called immune checkpoint inhibitors (CPIs). There are a large number of previous and ongoing clinical trials to investigate the role of CPIs in the treatment of a wide range of cancer types, signifying the importance of this class of immunotherapy [7,8,9,10]. Journal literature in the PubMed engine has been researched for the last decade to formulate the main aspects to be included in the review. In this article, we highlight the mechanism of action of major CPIs, their clinical impact, variation in effectiveness, response prediction, updated indications, current challenges and limitations, promising novel approaches, and future directions.

## 2. Immune Checkpoint Inhibitors (CPIs)

Immune checkpoints are classified as immune cell surface receptors that are involved in the control of activation or inhibition of immune response [11]. Immune checkpoint inhibitors (CPIs) represent a type of immunotherapy that boosts antitumor immune response by blockade of cell surface receptors of T lymphocytes [12]. This class of immunotherapy is considered the most thoroughly investigated to date and plays an important role in the treatment of multiple malignancies [13]. Two of the most promising checkpoint inhibition approaches that have been widely used in the last decade are blockade of PD-1/PD-L1 and CTLA-4 molecules [14]. Other targets such as inhibitory receptors T-cell immunoglobulin and mucin 3 (Tim-3), V-domain Ig suppressor of T-cell activation (VISTA), lymphocyte activation gene 3 (Lag-3), and activating molecules such as OX40 (CD134) and glucocorticoid-induced TNFR-related protein (GITR) are under investigation [15,16,17,18,19,20].

### 2.1. Development of CPIs

The discovery of T-cell-negative regulation by CTLA-4 was a spark to the establishment of CTLA-4 blockade as a form of cancer immunotherapy. The early work of Allison et al. has demonstrated that blocking CTLA-4 in mice was able to stop tumor formation, in addition to the establishment of immunological memory, which helped the mice to reject the tumor continuously. The success in preclinical models has led to the development of humanized monoclonal antibodies to inhibit the interaction of CTLA-4 with its ligand (B7) in patients with cancer and the establishment of clinical trials [21]. This was the start of a paradigm shift in the field of cancer immunotherapy by successfully activating the immune system toward tumors.

The discovery by Honjo et al. that the binding of PD-1/PD-L1 induces T-cell exhaustion inspired the idea of inhibiting this mechanism as a new strategy in cancer immunotherapy, which was worth further investigation [22]. Indeed, studies in preclinical models revealed enhanced T-cell activation and interaction in response to PD-L1 inhibition. In addition, similar blockade in mice tumor models showed elevated tumor-specific T-cell response and tumor regression [23]. The success in PD-1/PD-L1 inhibition in preclinical studies has encouraged the development of multiple humanized antibodies and the launch of clinical trials in patients with advanced cancers. The types of these antibodies and their indications are discussed further in the following sections.

### 2.2. CTLA-4 Inhibitors

**Figure 1:**
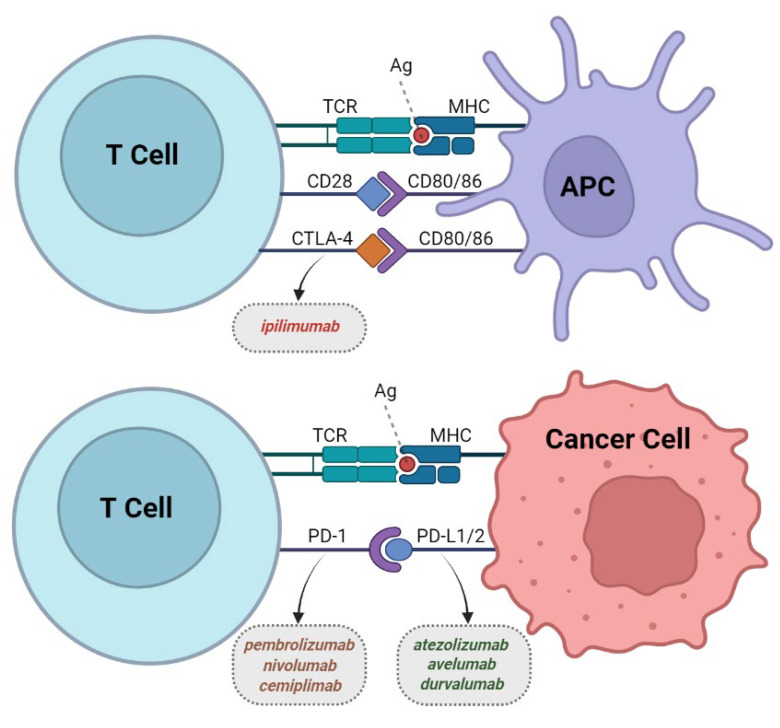
FDA-approved classes of immune checkpoint inhibitors (CPIs). CTLA-4 (through the interaction with its ligands B7-1/CD80 and B7-2/CD86) or PD-1 (via binding to its ligand PD-L1) trigger inhibitory signals to attenuate T-cell immune response. These T-cell receptor targets provide rationale for the use of CPIs such as anti-CTLA-4, PD-1, and PD-L1, which are illustrated with dotted-border boxes to increase immune response and kill tumor cells. CTLA-4: Cytotoxic T-lymphocyte-associated protein 4; PD-1/PD-L1: Programmed cell death protein-1 and its ligand-1, respectively; APC: Antigen-presenting cell; Ag: Antigen; TCR: T-cell receptor; MHC: Major histocompatibility complex. (Figure was designed with BioRender.com, https://help.biorender.com/en/articles/3619405-how-do-i-cite-biorender, accessed on 7 November 2022).

CTLA-4 is a coinhibitory member of the immunoglobulin superfamily that negatively regulates the activation of T cells through the interaction with its ligands B7-1 (CD80) and B7-2 (CD86) (Figure 1) [24]. CTLA-4 and CD28 have very similar protein sequences, and their genes are localized closely on chromosome 2q33 [25]. They both form homodimers and bind the same ligands, although with different affinities [26]. CTLA-4 binds with higher affinity and scavenges away the ligands from CD28, leading to negative signaling of T cells [27]. In the context of tumor immunoregulation, CTLA-4 acts in the early stage of T-cell response in the lymph nodes, as its ligands are mainly expressed on APCs [28]. This suggests that the absence of CTLA-4 can result in the unregulated proliferation of T cells, which was the insight to explore if CTLA-4 blockade may increase antitumor immune response.

There have been multiple studies that showed antitumor response by CTLA-4 blockade in animal models of breast, prostate, lymphoma, colon, and melanoma malignancies [29,30,31,32,33]. The increasing evidence of significant antitumor response, as shown in preclinical studies, has paved the way to investigate CTLA-4 blockade further in clinical trials. Ipilimumab, a human IgG1 monoclonal antibody (mAb) against CTLA-4, was the first treatment approved for melanoma in 2010 [34]. The treatment was associated with a significant increase in survival, durable response (>2.5 years), and potentially long-term control of the disease [35,36].

### 2.3. PD-1 Inhibitors

PD-1 is also a member of the immunoglobulin superfamily and plays a key coinhibitory role in the signaling of programmed cell death in response to the T-cell-mediated process [37]. It is more widely expressed than CTLA-4 and can be detected in multiple immune cell types within the tumor microenvironment (TME). During the binding of PD-1 to its ligand PD-L1 (B7-H1), it transmits an inhibitory signal that results in T-cell inhibition and eventually exhaustion (Figure 1) [38,39,40]. While CTLA-4 regulates T-cell activity at the priming phase, PD-1 mainly acts to limit T-cell activity in peripheral tissues at later stages of tumor growth [41].

Successful antitumor T-cell response to PD-1 inhibition has been shown in multiple models for colon cancer, melanoma, and pancreatic ductal adenocarcinoma [42,43]. This has stimulated multiple clinical trials that eventually led to FDA approval of pembrolizumab, nivolumab, then cemiplimab all as PD-1 inhibitors.

Pembrolizumab is a humanized IgG4 mAb that was initially approved by the FDA for the treatment of metastatic melanoma and non-small-cell lung cancer (NSCLC) [44,45]. In the subsequent years, the treatment was approved for a variety of tumor types, including squamous cell carcinoma of the head and neck (HNSCC) [46], solid tumors with high microsatellite instability (MSI-H) [47], advanced gastric cancer [48,49], cervical cancer [50,51], urothelial carcinoma [52], triple-negative breast cancer (TNBC) [53,54], tumors with high mutational burden (TMB-H) [55], and others (Table 1). This year, the FDA approved a new indication for pembrolizumab for MSI-H or mismatch repair-deficient (dMMR) advanced endometrial carcinoma as recommended by the KEYNOTE-158 trial [56]

Nivolumab is a fully human IgG4 mAb that was approved by the FDA in 2014 for the treatment of melanoma [57]. Consequently, new indications were approved for the treatment of NSCLC [58], renal cell carcinoma [59], Hodgkin’s lymphoma [60], HNSCC [61], hepatocellular carcinoma [62], esophageal squamous cell carcinoma [63], pleural mesothelioma [64], colorectal cancer (CRC) with dMMR or MSI-H [65], and others (Table 1). The year 2022 had multiple successful FDA approvals of nivolumab for multiple indications. Nivolumab has been approved in combination with LAG-3 inhibitor for the treatment of unresectable or metastatic melanoma based on results of the RELATIVITY-047 trial [66]. In addition, nivolumab in combination with chemotherapy was also approved as neoadjuvant therapy for early-stage NSCLC as per results of the CheckMate-816 study [67]. Moreover, nivolumab received dual approval for use in combination with either chemotherapy or ipilimumab for patients with unresectable advanced or metastatic esophageal squamous cell carcinoma (ESCC) as recommended by the CheckMate-648 trial [68].

Cemiplimab is a fully humanized IgG4 mAb that was the first FDA-approved drug in 2018 for the treatment of cutaneous squamous cell carcinoma (CSCC) [69]. The treatment showed better overall survival (OS) and progression-free survival (PFS) with an acceptable safety profile compared to EGFR inhibitors and chemotherapy [70].

### 2.4. PD-L1 Inhibitors

PD-1 can suppress activated immune cells via interaction with its ligands PD-L1 and PD-L2 [71]. PD-L1 (also known as B7-H1) is widely expressed on many tumor types and immune cells, whereas PD-L2 is mainly expressed on normal dendritic cells [17]. Tumors can exploit the PD-1/PD-L1 pathway to attenuate T-cell-mediated immunity, leading to abnormal proliferation of cancer cells. Our understanding of this interaction has made PD-L1 an attractive target for immunotherapy. There have been three FDA-approved PD-L1 inhibitors, which include atezolizumab, durvalumab, and avelumab.

Atezolizumab is a humanized IgG1 anti-PD-L1 mAb that was approved for the treatment of urothelial carcinoma in 2016 [72]. Due to the increased response rate, the indication was expanded later to include NSCLC [73,74], SCLC [75], melanoma [76], and hepatocellular carcinoma [77] (Table 1). Of note, the treatment was initially indicated for TNBC but now is no longer available as a treatment option because the objective of the IMpassion130 clinical trial was not met.

Durvalumab is an IgG1 anti-PD-L1 mAb that was first approved by the FDA for the treatment of urothelial bladder cancer [78]. A year later, the drug was approved for stage III NSCLC and extensive stage SCLC [79,80,81]. In 2022, the FDA approved durvalumab in combination with chemotherapy for patients with biliary tract cancer (BTC) based on the TOPAZ-1 clinical trial [82].

Avelumab is another fully human IgG1 anti-PD-L1 mAb that was approved by the FDA in 2017 and made a breakthrough to be the first treatment for metastatic Merkel cell carcinoma (MCC), a rare but aggressive form of skin cancer [83]. The treatment was then approved for patients with urothelial carcinoma and renal cell carcinoma [84,85].

There are noticeable advances in the process of developing new CPIs for multiple indications. Ongoing stage 3–4 clinical trials in the pipeline for CPIs are illustrated in Table 2. 

Although CPIs are a quickly advancing type of immunotherapy, there are multiple challenges and difficulties that can intimidate their efficacy. Primary resistance (when the tumor does not respond to the first-time administration of therapy) might happen in the case of CPI treatment [86]. In addition, there can be complications with acquired resistance, when the drug used before no longer works. This has been noticed in melanoma patients, where about 30% respond well at the beginning of the treatment, then they develop acquired resistance during the therapy course [87]. Another big limitation in the use of CPIs is the development of immune-related adverse events (irAEs). This type of adverse event can occur early or late during the treatment plan and manifests in different spectra and grades [88]. Furthermore, there are multiple biological factors that can directly or indirectly enhance or limit the CPI’s performance, and are discussed more in the following sections. These factors can be genomic (e.g., dMMR/MSI/TMB), immunological (e.g., TILs), microenvironmental (TME), and others.

## 3. Predictive Biomarkers for Clinical Response to CPIs

The use of CPIs has shown prominent success as a form of immunotherapy to treat various cancer types. However, only about 20–40% of patients receive evident benefits, which highlights the need to understand the variation in the treatment response and development of predictive biomarkers [89]. This step is necessary and valuable to select only those who are predicted to be responsive to treatment, and to minimize unnecessary costs and/or adverse side effects associated with treatment. There has been growing evidence that various biomarkers such as PD-L1 expression, tumor mutational burden (TMB), defective DNA mismatch repair (dMMR), microsatellite instability (MSI), tumor microenvironment (TME), and microbiota are associated with altered immunotherapy outcomes.

### 3.1. PD-L1 Expression

PD-L1 expression status on immune or tumor cells is considered one of the first biomarkers to predict response to CPIs [90]. Characterization of PD-L1 expression by immunohistochemistry (IHC) is a diagnostic test approved by the FDA and required prior to the treatment with PD-1 or PD-L1 inhibitors in multiple indications. Based on IHC, the patients are assigned a tumor proportion score (TPS), representing the percentage of tumor cells that express PD-L1. Currently, the focus is only on the expression of PD-L1 on tumor cells; however, there is growing evidence that it should be extended to include immune cells, as well to generate a combined positive score (CPS), which can identify additional subgroups with treatment benefit [91]. Multiple studies have shown a positive correlation between PD-L1 expression and response to CPIs. For example, it has been shown that NSCLC patients with TPS ≥ 50% were associated with significantly the longest PFS and OS (among the TPS groups) in response to pembrolizumab compared to chemotherapy [92,93]. In addition, patients with HNSCC and increased PD-L1 expression showed improved response to the same drug [46]. Nonetheless, PD-L1 expression alone remains an incomprehensive predictive marker, as there are still subgroups of negative PD-L1 patients who showed clinical benefit to CPIs [94]. Moreover, PD-L1 expression can vary markedly between different anatomic sites, during the clinical course, and IHC scoring [91,95], which confirms the need for additional and more comprehensive biomarker(s).

### 3.2. Tumor Mutational Burden (TMB)

The number of mutations (muts) per megabase (Mb) in cancer cells is known as tumor mutational burden (TMB) [96]. The cutoff for TMB to be considered high is variable based on tumor type (cutoffs of 10, 20, and >30 muts/Mb have been used in multiple studies) [55,97,98]. High TMB (TMB-H) has been used as an emerging biomarker of significant response to immune CPIs. A study in 2015 showed significantly improved objective response rate (ORR) and PFS in NSCLC patients with TMB-H in response to pembrolizumab [99]. Another study showed that nivolumab plus ipilimumab was significantly associated with longer PFS in NSCLC patients with TMB-H, and the response was maintained even if PD-L1 was not expressed [100]. In addition, the KEYNOTE-158 study, which mainly covered about 10 malignancies (mostly solid tumors), has revealed significant response of patients with TMB-H to pembrolizumab [101]. In response, the FDA has approved the same drug for all solid tumors with TMB-H (≥10 muts/Mb). While both PD-L1 expression and TMB are used as biomarkers of response to CPIs, they are not significantly correlated in most cancer types, and they seem to work in independent mechanisms to regulate the response [102]. Although there is some evidence to support that TMB might be a more predictive biomarker of response to CPIs than PD-L1 expression, the broad performance of TMB across all solid tumors is unclear and needs further investigation. For example, glioma patients with low TMB (TMB-L) but not TMB-H have shown favorable response to CPIs [103]. While TMB has made a significant milestone toward predicting response to CPIs, there is still an unmet need for a more reliable and comprehensive biomarker(s).

### 3.3. Defective DNA Mismatch Repair (dMMR) and Microsatellite Instability (MSI)

Intrinsic errors during DNA replication such as base mismatches and/or misincorporation are recognized and corrected by the DNA mismatch repair (MMR) system [104]. A defective MMR system can result in microsatellite instability (MSI), which is usually a hallmark of multiple cancers and characterized by evasion of apoptosis and accumulation of malignant mutations, which eventually promote tumorigenesis and neoantigen production [65,97]. dMMR/MSI subsequently results in increased immune cell filtration, which in turn makes the tumor cells more susceptible to CPIs [105]. Therefore, the role of dMMR/MSI as a predictive biomarker for response has been investigated in multiple studies. The significant response of patients dMMR and high MSI (MSI-H) to pembrolizumab in multiple tumor types and trials has consequently led to FDA approval of the drug for the treatment of solid tumors with the dMMR/MSI-H biomarker [106]. Similarly, a clinical trial reported that nivolumab plus ipilimumab has shown durable response in patients with dMMR/MSI-H colorectal cancer [65]. Of note, while some studies consider MSI as a specific type of MTB-H tumor, some evidence suggests that not all dMMR/MSI-H tumors are TMB-H. In fact, it has been shown that TMB-H tumors can exist without dMMR/MSI [105]. Thus, the strategy to utilize these biomarkers for the prediction of response to CPIs needs further optimization. In addition, with the common belief that many MMR-deficient tumors are more responsive to CPI treatment, there is now a convincing rationale to investigate the clinical benefit of CPIs in combination with MMR inhibitors. This form of treatment could convert immunogenically “cold” tumors into “hot”, thus making them more responsive to therapy.

### 3.4. Tumor Microenvironment (TME)

The reservoir of blood vessels, immune cells, and molecules that surround a tumor form an ecosystem called the tumor microenvironment (TME). Our increased understanding of the TME has been very crucial in advancing the field of immune-oncology and understanding the multiple mechanisms involved in the modulation of response to immunotherapy. Tumors now can be classified into multiple groups based on their “immune contexture”. For example, tumors with a high level of infiltrating lymphocytes (TILs) are considered immunogenically “hot”, while those with low TILs are considered immunogenically “cold” [107]. Based on that, transforming environments of tumors from “cold” into “hot” environments can be a key to increase response to CPIs and is currently under investigation.

Immune cell infiltration has shown high correlation with response to CPIs in patients with colorectal cancer and indicated higher prediction than MSI [108]. The systematic characterization of CD3+ and CD8+ T cells (immunoscore) is now considered a tool to predict prognosis and response to CPIs [109]. In addition to colorectal tumors, CD8+ TILs are generally associated with promising outcomes in breast cancer and HCC [110,111]. However, given that the composition of TME is tremendously variable, CD8+ TILs cannot be solely the biomarker for response to CPIs in the TME, and future approaches should look into collective signatures from several TME components. These can include other T-cell markers such as TCF7+, CD4+, FoxP3+, and IFNγ; B-cell lymphoid structures; innate immune cells; and stromal markers [112].

### 3.5. Microbiota

The collective genomes of microbes (including bacteria, viruses, fungi, and others) that inhabit the gut are called the gut microbiome. On the other hand, the term microbiota represents the microorganisms that inhabit that specific environment. By influencing the immune system, microbiota have shown to alter the efficacy of response to vaccines and cancer therapy, including CPIs [113,114]. The mechanisms by which microbiota induce immune response can include increased secretion of IL-2 and IFN, and T-cell activation, leading to direct targeting of cancer cells [115]. Loss of proper microbial diversity (also known as dysbiosis) has been linked to poor response to CPIs [116]. Quite the reverse, enrichment of specific species such as *Bifidobacterium*, *Faecalibacterium*, *Lactobacillus*, *Akkermansia*, and others was associated with better response to CPIs in multiple studies, including patients with melanoma and HCC [117]. Of note, fecal microbiota transplantation (FMT) of “responders” gut flora into germ-free mice improved control of tumor growth and efficacy of CPIs [118]. This has provided a strong push toward clinical translation, and there are ongoing trials to treat CPI-resistant patients with “responders” MT (e.g., NCT03353402 and NCT03341143). Taken together, these findings highlight the importance of taking factors that alter the microbiota diversity prior to treatment with CPIs into consideration. Such factors can include antibiotics, radiation therapy, diet, and other drugs.

## 4. Toxicity and Management of Adverse Effects

Even though they are considered among the most successful forms of immunotherapy, CPIs have been associated with immune-related adverse events (irAEs). irAEs can vary depending on multiple factors, such as the type of CPIs used, tumor site, and patient susceptibility. The toxicity can be systemic, dermatological, gastrointestinal, and endocrinal, although skin and colon are the most frequently affected organs [119]. A higher frequency of toxicity profiles has been associated with anti-CTLA-4 agents compared to anti-PD-1/PD-L1, probably due to the fact that the CTLA-4 response is at the early stage of T-cell activation. irAEs have been reported in 27% vs. 16% with the use of anti-CTLA-4 (ipilimumab) and anti-PD-1 (nivolumab), respectively, and the frequency increased to 55% when both drugs were used in combination [120]. The most common adverse effect linked to CPIs is fatigue, which is, luckily, not a major factor to limit treatment duration nor requires medical intervention. The incidence of fatigue can vary from 16% to 24% to 40% with the use of anti-PD-1, anti-PD-L1, and anti-CTLA-4, respectively [36,121]. A meta-analysis of 2540 records of PD-1/PD-L1 inhibitor-based treatment adverse events have been performed to evaluate toxicity profiles [122]. The most commonly reported adverse events are summarized in Table 3.

Although less frequent, life-threatening irAEs exist and can include *de novo* insulin-dependent diabetes, pituitary dysfunction, inflammatory pneumonitis, renal nephritis, eye damage, and even mortality. Furthermore, the data regarding CPI use in children is limited; thus, there is an increased concern of unpredictable off-target effects on vital organs given that children might have less mature organs.

Of note, recent data from a multicenter study on spectrum and grade of irAEs has shown that although less common, late irAEs (i.e., after 12 months of CPIs treatment) are fairly common in long responders, and they occur with different manifestations [88]. These findings highlight the importance of the evolution depiction of toxicity over time.

Depending on the severity, the manifestation of irAEs usually requires the administration of corticosteroids as early as possible to prevent permanent damage. For serious irAEs, corticosteroids can be coupled with tumor necrosis factor (TNF) inhibitors and immunosuppressants such as mycophenolate mofetil [123,124]. The data on the impact of corticosteroids on the efficacy of CPIs have been controversial. Although there is evidence that supports the association of corticosteroids with worse outcomes in patients with NSCLC [125] and melanoma [126], there was no association in other studies, and the patients maintained durable response [127,128]. Further investigation is needed to better elucidate the impact of corticosteroids on CPIs efficacy.

In 2021, the American Society of Clinical Oncology (ASCO) published an update on the management of irAEs in patients treated with CPIs based on a systematic review of 175 studies from 2017 to 2021 [129]. Their recommendations are summarized in Table 4.

## 5. Novel Delivery Approaches

CPIs have been a powerful immunotherapeutic clinical strategy to treat a wide range of cancer types. Nonetheless, the unpredictable adverse effects, which sometimes can be severe and life threatening, represent a major challenge. There is an increasing number of novel approaches to improve the delivery and accumulation of CPIs into the tumor site, eventually leading to reduced off-target effect and enhanced efficacy. These can involve the use of nanoparticles, molecular conjugates, biomaterials, viral vectors, and bacteria (Table 5).

In addition to being known as delivery systems, nanoparticles can be exploited to deliver combinations of therapeutic agents, to both reduce off-target effects and convert the TME from immunogenically “cold” to “hot”. The use of lipid-protamine-DNA (LPD) nanoparticles loaded with PD-L1 plasmid (to inhibit PD-L1 signaling) in addition to oxaliplatin (to induce immune response) in mice has been shown to work synergistically to inhibit tumor growth, reduce toxicity, and may cause the tumor to be more immunogenic, leading to better response to PD-L1 therapy [130]. Another study used antibody-targeted nanoparticles to deliver the TGFβR1 inhibitor or TLR7/8 agonists to target PD-1-expressing T cells both in circulation and in the TME, respectively. Subsequently, this increased mice survival, CD8+ TILs, and sensitized the tumor to anti-PD-1 therapy [131]. Moreover, a reporter nanoparticle with PEG-conjugated PD-L1 antibody revealed enhanced antitumor activity in lung and breast cancer preclinical models [132]. These successful studies warrant further exploration of the use of nanoparticles, especially at the clinical trial level, potentially for improved efficacy and reduced toxicity.

Matrix-binding molecular conjugates are another idea for intra- and peritumoral delivery to reduce systemic adverse effects and prolong the retention of CPIs [133]. In that strategy, CPIs were conjugated to placental growth factor 2 (PIGF2) peptide, which has high affinity to multiple extracellular matrix (ECM) proteins. The localization of PIGF2-CPIs significantly reduced treatment-associated toxicity, delayed tumor growth, and improved survival in murine models of melanoma and breast cancer. Such an approach might be doable to target unreachable tumors by the local delivery (e.g., intraperitoneal injection) of CPIs to reduce toxicity and boost efficacy [134].

The application of engineered biomaterials has made perceptible progress in the localization of CPIs and immunotherapy. The biomaterials can be implantable, injectable, or transdermally administered. Implantable biomaterials (e.g., the use of poly lactide-co-glycolide (PLG) as a scaffold to release bioactive substances) have been investigated in cytokines, vaccines, and T-cell engineering forms of immunotherapy [13]. Injectable biomaterials, such as hydrogels, have the ability to form a cross-linked network, which helps prevent the degradation of encapsulated CPIs. One study used a fibrin hydrogel to load cyclophosphamide (CTX), which is a small chemotherapeutic agent, in addition to anti-PD-L1 in breast and ovarian tumor models [135]. Given its small size, CTX was released first to make the TME more immunogenic, which maximized the efficacy of anti-PD-L1 that was slowly released thereafter. Another study exploited the fact that reactive oxygen species (ROS) are elevated in the TME and are associated with immunosuppression [136]. An injectable hydrogel into melanoma and breast tumor models was biodegraded in response to ROS to release its content of anti-PD-L1 and the chemotherapeutic agent gemcitabine. This eventually led to increased immunogenicity in the TME, reduced tumor growth, and improved survival. Minimally invasive transdermal delivery using microneedles has been designed for localized transport of CPIs. In one example, hyaluronic acid (HA) and anti-PD-1 were encapsulated in pH-sensitive nanoparticles, which, when in the acidic TME, their content of anti-PD-1 can be released [137]. This strategy induced robust immune response and improved survival in a melanoma mouse model.

On a genetic level, the delivery of CPIs can be accomplished by multiple viral vector systems such as retroviral, adenoviral, and oncolytic viral vectors [134]. In one approach, a retroviral-replicating vector (RRV) was designed to express anti-PD-L1 microRNA (RRV-miRPDL1) to downregulate PD-L1 in human cancer cell lines [138]. The results revealed sustained inhibition of PD-L1 protein expression by more than 75%. Furthermore, assessment of the immunologic effect showed restoration of CD8+ T cells in a similar fashion to that seen by antibody blockade of PD-L1. Another example is packaging the coding sequence of anti-PD-1 into tumor-targeted Her2/neu-specific adeno-associated virus (AAV) vectors [139]. Unfortunately, that approach showed modest tumor growth reduction with no significant changes in the levels of anti-PD-1 in the tumor. Oncolytic viruses (OVs) selectively infect, replicate, and lyse cancer cells while leaving normal cells intact. The combination of OVs and CPIs is being investigated by multiple clinical trials in patients with melanoma, CRC, HCC, RCC, NSCLS, and others, which forms an exciting new generation of cancer treatment [140]. This combination has demonstrated a significant synergistic effect in metastatic and local tumors.

Because of their favored colonization of tumor sites, bacteria represent a natural vehicle for the local delivery of CPIs. One study engineered a probiotic strain of *E*. *coli* for intratumor release of nanobodies against PD-L1 and CTLA-4 into a mouse model of CRC in a controlled manner [141]. This attractive delivery modality stimulated systemic antitumor response with significantly reduced toxicity. Although the use of bacteria as a delivery strategy for CPIs has been very limited, it represents a great opportunity to advance cancer immunotherapy that is worth further exploration.

## 6. Conclusions

The introduction of CPIs in the last decade has undoubtedly revolutionized the field of cancer immunotherapy with remarkable clinical efficacy. Undeniably, CPIs have improved treatment for a broad spectrum of solid tumors such as melanoma, NSCLC, renal carcinoma, and several cancer types. Even more, CPIs have established a new vision of dynamic cancer management where molecular biomarkers can be used to predict patient response and level of toxicity. New tools such as PD-L1 expression, TMB, dMMR/MSI, TME, and microbiota composition can now be used to inform treatment decisions effectively. Nevertheless, the improved overall survival is complicated by the manifestation of irAEs, and there is a great proportion of patients treated with CPIs but without significant benefit from therapy. With the rapid growth of CPI clinical use, complexity of the immune system, and huge variation in the TME of different cancer types, the need for comprehensively effective biomarker(s) for the prediction of response and toxicity becomes increasingly necessary. The future will depend upon the identification of multiple biomarker signatures that are specific to each clinical setting. Furthermore, the development of better drug combinations and delivery approaches with enhanced efficacy and reduced toxicity to potentiate clinical activity is essential.

## Figures and Tables

**Table 1:** FDA-approved immune CPIs and their indication(s).

CPI Target	Drug	Class	1st Approval Year	Indication(s)
CTLA-4	Ipilimumab	IgG1 mAb	2010	Melanoma.
PD-1	Pembrolizumab	IgG4 mAb	2016	Melanoma, NSCLC, HNSCC, MSI-H solid tumors, GC, cervical cancer, urothelial carcinoma, TNBC, TMB-H/dMMR endometrial carcinoma.
	Nivolumab	IgG4 mAb	2014	Melanoma, NSCLC, RCC, Hodgkin’s lymphoma, HNSCC, HCC, ESCC, pleural mesothelioma, MSI-H/dMMR CRC.
	Cemiplimab	IgG4 mAb	2018	Cutaneous squamous cell carcinoma.
PD-L1	Atezolizumab	IgG1 mAb	2016	Urothelial carcinoma, NSCLC, SCLC, melanoma, HCC.
	Durvalumab	IgG1 mAb	2017	Urothelial bladder cancer, NSCLC, SCLC, BTC.
	Avelumab	IgG1 mAb	2017	Merkel cell carcinoma, urothelial carcinoma, RCC.

Abbreviations: CTLA-4 = Cytotoxic T-lymphocyte-associated protein 4; PD-1/PD-L1 = Programmed cell death protein-1 and its ligand-1, respectively; IgG = Immunoglobulin G; NSCLC = Non-small-cell lung cancer; HNSCC = Squamous cell carcinoma of the head and neck; GC = Gastric cancer; TNBC = Triple-negative breast cancer; RCC = Renal cell carcinoma; HCC = Hepatocellular carcinoma; ESCC = Esophageal squamous cell carcinoma; CRC = Colorectal cancer, SCLC = Small-cell lung cancer; BTC = Biliary tract cancer; TMB-H = Tumors with high mutational burden; MSI-H = high microsatellite instability; dMMR = Mismatch repair deficient.

**Table 2:** Ongoing stage 3 or 4 active clinical trials that involve CPIs.

Rank	NCT Number	Title	Acronym	Status	Conditions	Interventions	Phase	Location(s)
1	5335928	Abatacept in Immune Checkpoint Inhibitor Myocarditis	ATRIUM	Recruiting	Myocarditis Acute, cancer	Drug: abatacept plus, drug: placebo	3	United States
2	4338269	A Study of Atezolizumab in Combination with Cabozantinib Compared to Cabozantinib Alone in Participants With Advanced Renal Cell Carcinoma After Immune Checkpoint Inhibitor Treatment	CONTACT-03	Active, not recruiting	Carcinoma, renal cell	Drug: atezolizumab, drug: cabozantinib	3	United States, Argentina, Australia, Canada, Denmark, France, Germany, Greece, Italy, Japan, Korea, Poland, Russian Federation, Russian Federation, Spain, United Kingdom
3	2654587	Study of OSE2101 Versus Standard Treatment as 2nd or 3rd Line in HLA-A2 Positive Patients with Advanced NSCLC After Failure of Immune Checkpoint Inhibitor	ATALANTE 1	Active, not recruiting	Non-small-cell lung cancer	Drug: OSE2101, drug: docetaxel, drug: pemetrexed	3	United States, Czechia, France, Germany, Hungary, Israel, Italy, Poland, Spain, United Kingdom
4	4987203	Study to Compare Tivozanib in Combination with Nivolumab to Tivozanib Monotherapy in Subjects With Renal Cell Carcinoma	NA	Recruiting	Renal cell carcinoma	Drug: tivozanib, drug: nivolumab	3	United States, Argentina, Australia, Belgium, Brazil, Canada, Chile, Czechia, France, Germany, Italy, Mexico, Poland, Portugal, Spain, United Kingdom
5	3469960	Double Immune Checkpoint Inhibitors in PD-L1-positive Stage IV Non-Small Lung Cancer	DICIPLE	Active, not recruiting	Non-small-cell lung cancer metastatic	Drug: ipilimumab, drug: nivolumab	3	France
6	4590963	Assessment of Efficacy and Safety of Monalizumab Plus Cetuximab Compared to Placebo Plus Cetuximab in Recurrent or Metastatic Head and Neck Cancer	INTERLINK-1	Active, not recruiting	Squamous cell carcinoma of the head and neck	Drug: monalizumab, drug: cetuximab	Phase 3	United States, Argentina, Australia, Belgium, Brazil, Bulgaria, Canada, France, Germany, Greece, Italy, Japan, Korea, Netherlands, Philippines, Portugal, Russian Federation, Spain, Switzerland, Taiwan, United Kingdom
7	5106335	A Study to Evaluate Camrelizumab Combined with Famitinib as Subsequent Therapy in Patients With Advanced NSCLC	NA	Recruiting	Advanced NSCLC	Drug: camrelizumab + famitinib, drug: famitinib, drug: docetaxel	3	China
8	4059887	Evaluation of Blood TMB for the Efficacy of Atezolizumab [BUDDY]	BUDDY	Recruiting	Lung neoplasm Malignant	Drug: atezolizumab injection (Tecentriq)	4	Korea
9	3427827	PD-1 Antibody Versus Best Supportive Care After Chemoradiation in Locoregionally Advanced Nasopharyngeal Carcinoma	PACIFIC-NPC	Recruiting	Nasopharyngeal neoplasms	Drug: camrelizumab	3	China
10	4907370	PD-1 Blockade Combined with De-intensification Radical Chemoradiotherapy in Nasopharyngeal Carcinoma	TIRA	Recruiting	Nasopharyngeal carcinoma	Drug: PD-1 blocking antibody, drug: gemcitabine, drug: cisplatin (80 mg/m^2^), drug: cisplatin (100 mg/m^2^), radiation: intensity-modulated radiotherapy	3	China
11	4334759	Durvalumab With Chemotherapy as First Line Treatment in Advanced Pleural Mesothelioma	DREAM3R	Recruiting	Mesothelioma, pleural mesothelioma, malignant pleural mesothelioma	Drug: durvalumab, drug: standard chemotherapy, drug: ipilimumab and nivolumab	3	United States, Australia, New Zealand
12	4799249	Trilaciclib, a CDK 4/6 Inhibitor, in Patients Receiving Gemcitabine and Carboplatin for Metastatic Triple-Negative Breast Cancer (TNBC)	PRESERVE 2	Active, not recruiting	Triple-negative breast cancer (TNBC), breast cancer	Drug: trilaciclib, drug: placebo, drug: gemcitabine, drug: carboplatin	3	United States, Australia, Bulgaria, China, France, Georgia, Moldova, Poland, Russian Federation, Serbia, Spain, Ukraine
13	2973789	Effect of Tumor Treating Fields (TTFields) (150 kHz) Concurrent with Standard of Care Therapies for Treatment of Stage 4 Non-Small Cell Lung Cancer (NSCLC) Following Platinum Failure (LUNAR)	NA	Active, not recruiting	Non-small-cell lung cancer (NSCLC)	Device: NovoTTF-200T, drug: immune checkpoint inhibitors or docetaxel	3	United States, Austria, Belgium, Bulgaria, Canada, China, Czechia, France, Germany, Hong Kong, Italy, Netherlands, Poland, Serbia, Spain, Switzerland

Data obtained via https://clinicaltrials.gov, accessed on 18 December 2022.

**Table 3:** Most commonly reported adverse events linked to PD-1 or PD-L1 inhibitor-based combination therapies (summarized from Zhou et al. 2021 [122]).

Adverse Event Grade	Adverse Event	Frequency %, [95% Confidence Interval]
All-Grade	Anemia	45 [32.4–59.1]
	Fatigue	34.3 [27.5–41.9]
	Dysphagia	30 [18.7–44.5]
Grade 3 or Higher	Neutropenia	19.6 [13.5–27.7]
	Hypertension	9.3 [5.7–14.9]
	Lipase increase	7.2 [5.2–9.9]
	Lymphopenia	10.3 [4.5–21.8]

**Table 4:** Summary of ASCO recommendations on irAEs management in patients treated with CPIs.

Grade	Management
1	CPI therapy should be continued with close monitoring for grade 1 toxicities, except for some neurologic, hematologic, and cardiac toxicities.
2	CPIs therapy may be suspended for most grade 2 toxicities, with consideration of resuming when symptoms revert ≤ grade 1.
3	Generally, warrant suspension of ICPs and the initiation of high-dose corticosteroids (should be tapered over the course of at least 4–6 weeks).
4	In general, permanent discontinuation of ICPis is recommended with grade 4 toxicities, except for endocrinopathies that have been controlled by hormone replacement.

Additional information is available at www.asco.org/supportivecare-guidelines.

**Table 5:** Summary of selected novel delivery strategies for immune CPIs.

Approach	Advantages	Limitations
Nanoparticles	Stimulate tumor immunogenicity, localized delivery, cargo protection.	High manufacturing cost, design complexity, possible toxicity, efficacy needs further characterization.
Matrix-binding molecular conjugates	Prolonged retention, localized delivery, efficient targeting of ECM.	Targeting restricted ECM can be challenging, ECM expression in healthy tissue may cause off-targeting and toxicity.
Engineered biomaterials	Adjustable design, localized delivery, drug controlled-release,	Acute/chronic inflammatory reactions, biocompatibility and biodegradability are not fully characterized, difficulties with hydrophobic drugs.
Viral vector systems	Multiple clinical trials using OVs, easily genetically modifiable, high level of expression.	Antiviral response can be a limiting factor, need complex design for improved efficacy, high regulatory standards.
Bacteria	Natural colonization of tumor sites, easily genetically engineered, potential for oral delivery (noninvasive).	The approach still in infancy, issues with microtumors, can be difficult to apply in immunocompromised individuals.

Abbreviations: ECM = Extracellular matrix; OVs = Oncolytic viruses.

## Data Availability

Not applicable.
